# Pharmacogenetic variants in *TPMT* alter cellular responses to cisplatin in inner ear cell lines

**DOI:** 10.1371/journal.pone.0175711

**Published:** 2017-04-13

**Authors:** Amit P. Bhavsar, Erandika P. Gunaretnam, Yuling Li, Jafar S. Hasbullah, Bruce C. Carleton, Colin J. D. Ross

**Affiliations:** 1 Faculty of Pharmaceutical Sciences, The University of British Columbia, Vancouver, British Columbia, Canada; 2 BC Children’s Hospital Research Institute, Vancouver, British Columbia, Canada; 3 Division of Translational Therapeutics, Department of Pediatrics, Faculty of Medicine, The University of British Columbia, Vancouver, British Columbia, Canada; 4 Department of Medical Genetics, Faculty of Medicine, The University of British Columbia, Vancouver, British Columbia, Canada; University of South Alabama Mitchell Cancer Institute, UNITED STATES

## Abstract

Cisplatin is a highly-effective and widely-used chemotherapeutic agent that causes ototoxicity in many patients. Pharmacogenomic studies of key genes controlling drug biotransformation identified variants in thiopurine methyltransferase (*TPMT*) as predictors of cisplatin-induced ototoxicity, although the mechanistic basis of this interaction has not been reported. Expression constructs of TPMT*3A, *3B and *3C variants were generated and monitored in cultured cells. Cellular TPMT*3A levels were detected at >20-fold lower amounts than the wild type confirming the unstable nature of this variant. The expression of wild type *TPMT* (TPMT*1) in two murine ear cell lines, HEI-OC1 and UB/OC-1, significantly mitigated their susceptibility to cisplatin toxicity. Cisplatin treatment induced *Tlr4* gene expression in HEI-OC1 cells and this response was blunted by the expression of wild type *TPMT* but not TPMT*3A. In line with the significant mitigation of TPMT*1-expressing cells to cisplatin cytotoxicity, these findings demonstrate a drug-gene interaction between increased TPMT activity and decreased susceptibility to cisplatin-induced toxicity of inner ear cells.

## Introduction

Cisplatin is a chemotherapeutic that is widely used in patients to treat solid tumours owing to its high effectiveness[1, 2]. However, cisplatin use is constrained by irreversible adverse drug reactions, including ototoxicity, which manifests as permanent, bilateral sensorineural hearing loss in 40–60% of treated children, and 20–40% of adults, although some estimates range up to 90% in children depending on assessment criteria[3–8]. At a cellular level, cisplatin-induced ototoxicity is characterized by reactive oxygen species formation, ensuing cellular damage and apoptosis in inner and outer hair cells of the ear including the Organ of Corti[4, 9].

The inter-individual range of susceptibility to cisplatin-induced ototoxicity is broad and poorly predicted by clinical factors alone[10, 11]. Genetic factors associated with cisplatin-induced ototoxicity have been reported, many in genes associated with reactive oxygen species detoxification and drug absorption, metabolism, distribution and excretion (ADME)[12]. One such pharmacogenomic association was identified, and subsequently replicated, in two independent cohorts of Canadian children (approximately 300 cisplatin-treated children)[13, 14]. The underlying genomic variation is linked to the *3A haplotype of the *TPMT* gene, resulting in an 8.9 odds ratio of developing cisplatin-induced ototoxicity[13]. Since the initial replication of the TPMT-cisplatin-induced ototoxicity pharmacogenomic association[13], an independent group observed a similar association in a cohort of Spanish children[15]. By contrast, this association was not replicated in other cohorts[15, 16], that were treated with vastly different protocols compared to the original studies. For example, confounding treatments such as craniospinal irradiation and otoprotectant treatments (amifostine) were used for most patients, which could override genetic predispositions[17]. Notably, in a patient cohort that was similarly treated to those of the original studies, the genetic association with *TPMT* was preserved, albeit in a small cohort of patients that was underpowered to detect meaningful associations.

*TPMT* encodes thiopurine *S*-methyl transferase, an enzyme that catalyzes the transfer of a methyl group from the methyl donor *S*-adenosylmethionine (SAM) to the sulphur residue of thiopurines, such as 6-mercaptopurine and azathioprine for their metabolism and detoxification. Pharmacogenetic variants in TPMT have been associated with severe life-threatening myelosuppression associated with the use of the immunosuppressant azathioprine and other thiopurines[18]. Well characterized variants in TPMT include TPMT*3B (rs1800460, minor allele frequency 1.3%) that encodes the nucleotide change 460C>T, corresponding to the non-synonymous coding change A154T; TPMT*3C (rs1142345, minor allele frequency 4%) that encodes the nucleotide change 719T>C, corresponding to the non-synonymous coding change Y240C; and TPMT*3A which contains both TPMT*3B and *3C variants. Given that thiopurines are direct substrates of TPMT, the mechanistic basis of the pharmacogenetic association with azathioprine differs from that with cisplatin, where a direct interaction with cisplatin has not been demonstrated. Thus, to explore a potential drug-gene interaction between cisplatin and *TPMT* we sought to examine the effect of TPMT deficiency on cisplatin phenotypes in model inner ear cell lines. Our findings establish that there is a drug-gene interaction between cisplatin and *TPMT in vitro*.

## Materials and methods

### Cells, constructs and reagents

HEK293T cells were purchased from ATCC (Cedarlane), and cultured in DMEM supplemented with 10% FBS, 100 U/ml Penicillin, 100 μg/ml Streptomycin and grown at 37°C in the presence of 5% CO_2_. The murine inner ear cell line HEI-OC1 (ref. [19]) was obtained from the laboratory of Dr. Federico Kalinec (UCLA) in April 2014 and were cultured in DMEM supplemented with 10% FBS, 100 U/ml Penicillin, 100 μg/ml Streptomycin. The murine inner ear cell line UB/OC-1 (ref. [20]) was obtained from the laboratory of Dr. Leonard Rybak (SIU) in August 2012 and were cultured in RPMI supplemented with 10% Fetal Clone II. Murine inner ear cells were grown at 33°C in the presence of 10% CO_2_. All cell lines screened negative for mycoplasma contamination. Transfections for gene expression were performed using Effectene (Qiagen) or X-tremeGENE 9 (Roche), and for *Tpmt* silencing were performed with Dharmafect 1 reagent (Thermofisher) according to the manufacturer’s specifications. An expression clone for *TPMT* was purchased from Origene (pCMV6-XL5*TPMT*). For gene expression analyses, RNA was purified with the Ambion Purelink RNA mini kit using Purelink homogenizers and Purelink on-column DNAse digestion and cDNA was generated using the Invitrogen Superscript III first strand synthesis kit according to the manufacturer’s specifications (Life Technologies). Real-time RT-PCR (qPCR) experiments of murine *Tpmt* and *Hprt1* gene expression in HEI-OC1 cells was performed using primer pairs *Tpmt-*for2q/*Tpmt-*rev2q and *Hprt1-*for1q/*Hprt1-*rev1q, respectively. The primer sequences used in this study are listed in S1 Table. qPCR was performed on the PikoReal 96 Real-Time PCR system (Thermo Scientific) and relative gene expression was calculated by the ΔΔC_q_ method using the instrument software. The rat anti-HA 3F10 monoclonal antibody (Sigma-Aldritch, cat# 11867423001; Antibody Registry AB_10094468) and the mouse anti-GAPDH 6C5 monoclonal antibody (Millipore, cat# MAB374; Antibody Registry AB_2107445) were used at (1:1,000) dilution, while IRDye 800CW Goat anti-Rat and IRDye 800CW Goat anti-Mouse polyclonal antibodies (Mandel Scientific, cat# 925–32219 and 925–32210) were used at (1:5,000) dilution.

### Cloning

The wild type *TPMT* (*1) open reading frame (ORF) was PCR amplified from pCMV6-XL5*TPMT* (Origene) using *TPMT-*for1 and *TPMT-*rev1 primers. The amplified product was TOPO-cloned into PCR4-TOPO according to the manufacturer’s specifications (Invitrogen). Sanger sequencing revealed the *TPMT* ORF contained the *1S variant. Site directed mutagenesis was performed using the Quikchange II reagent kit (Agilent) according to the manufacturer’s specifications. The *3B variant was created using primer pair *TPMT*g460a_2/*TPMT*g460a_as2 (also reverts *1S to *1). The *3A variant was created using the TPMT*3B template and primer pair *TPMT*a719g/*TPMT*a719g_as. The *3C variant was also separately created using primer pair *TPMT*a719g/*TPMT*a719g_as and a TPMT*1 template that was itself created by reversion of the *1S variant using primer pair *TPMT*t474c /*TPMT*t474c_as.

### *Tpmt* gene silencing

*Tpmt* expression was silenced in UB/OC-1 cells using 50 nM siGENOME mouse *Tpmt* SMARTpool siRNA or siGENOME Non-Targeting siRNA #3 (Dharmacon). siRNA transfections were performed using Dharmafect 1 transfection reagent (Dharmacon) for 24 hours according to the manufacturer’s specifications. *Tpmt* expression was monitored by SYBR Green qPCR conducted in a 10 μl reaction consisting of 5 μl PowerSybr Green Master Mix, 2 μl of cDNA, and 300 nM primers using cycling conditions: 95°C—7 min and 40 cycles of 95°C—5 sec, 60°C—30 sec. Under these conditions *Tpmt* expression was decreased by 55% in UB/OC-1 cells (S1 Fig).

### TPMT protein quantification

TPMT expression constructs were transfected into HEK293T or HEI-OC1 cells and lysate was generated 48 hours post-transfection. 40 μg of lysate was separated on a pre-cast 4–20% TGX gradient gel (BioRad) and then transferred to nitrocellulose using the mixed MW preset on the Transblot Turbo (BioRad). Probed membranes were scanned on the Odyssey imaging system (LI-COR). Membranes were stripped with Reblot Strong Stripping Buffer (Millipore) according to the manufacturer’s specifications.

### Cell viability assay of cisplatin-treated cells

5 x 10^3^ HEI-OC1 or UB/OC-1 cells were seeded into each well of a 96-well plate in the appropriate medium indicated above and grown overnight at 33°C and 10% CO_2_. The following day cells were transfected with TPMT*1 and TPMT*3A expression constructs. UB/OC-1 cells were transfected with siRNA to silence endogenous *Tpmt* as indicated above, 24h prior to transfection of *TPMT* expression constructs. The next day cells were treated with 0, 0.316, 1, 3.16, 10, 31.6, 100 or 316 μM cisplatin for 48 hours. Cell viability was assayed using MTT (Sigma) and absorbance was read on a POLARstar Omega plate reader (BMG Labtech).

### Colony survival assay of cisplatin-treated cells

2 x 10^6^ HEI-OC1 cells were seeded into a 10 cm dish in DMEM media supplemented with 10% FBS and 1% L-glutamine and grown overnight at 33°C and 10% CO_2_. The following day cells were transfected with TPMT*1 and TPMT*3A expression constructs as indicated above. The next day cells were harvested and reseeded at 6 x 10^5^ cells/well in 6-well plate and treated with 3.16, 31.6, or 316 nM cisplatin for 48 hours. Cells were then harvested, pooled with culture supernatants and PBS washes, and reseeded at 200 cells per well in a 24 well plate and grown at 33°C and 10% CO_2_ for 1–2 weeks until colonies were visible. Colonies were methanol-fixed, stained with Cresyl Violet then counted and imaged on an Evos XL Core microscope. Two independent experiments were performed in duplicate.

### Gene expression analyses

2 x 10^5^ HEI-OC1 cells were seeded into each well of a 6-well dish in DMEM media supplemented with 10% FBS and 1% L-glutamine. The following day fresh medium containing 0, 10 and 25 μM cisplatin was added to cells. Cells were grown for 24 hours then total RNA was immediately purified and used for cDNA synthesis. qPCR reactions were conducted in a 10 μl reaction volume that consisted of 5 μl 2X TaqMan Universal Master Mix, 0.5 μl TaqMan probe and 2 μl cDNA with standard cycling conditions on PikoReal instrumentation (Thermo) according to the manufacturer’s specifications. Validated Taqman assays were used to detect expression of murine Tlr4 (Mm00445273_m1) and Hprt1 (Mm00446968_m1).

### Data analyses

Cell viability data was analyzed using GraphPad Prism v5 and fitted to a non-linear regression, dose-response log (inhibitor) vs. normalized response model. Statistical significance of IC_50_ values was analyzed using the extra sum-of-squares F test. Relative gene expression was calculated using *Hprt1* as a housekeeping gene using PikoReal software. Western blot quantification was performed using ImageLite software (LI-COR).

## Results

**Fig 1:**
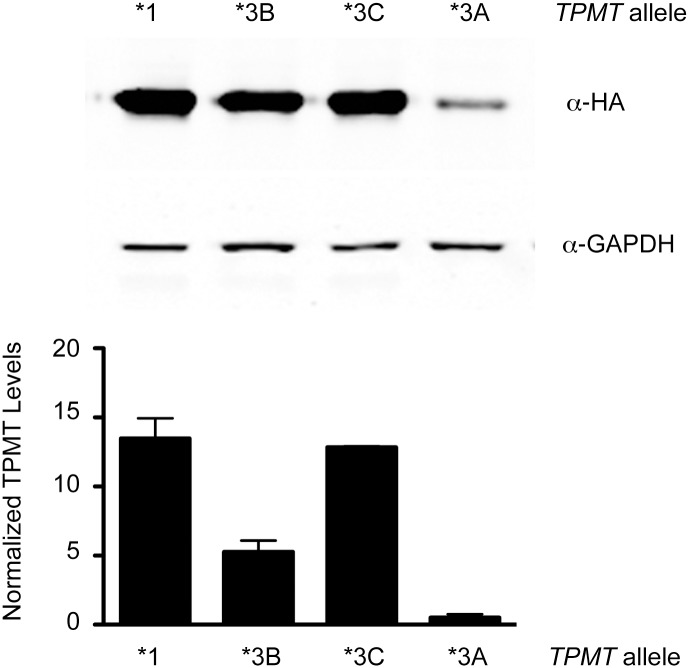
Pharmacogenetic variants in TPMT show differential stability in cell culture. *Top*, Representative western blot of HEK293T cells expressing indicated *TPMT* variants. TPMT was specifically detected using an HA-epitope tag. *Bottom*, quantification of West blot analysis, normalizing HA signal intensity to corresponding GAPDH signal intensity. Shown are results from two independent experiments. TPMT*3A levels are 4% of wild type TPMT levels.

To study the role of *TPMT* pharmacogenetic variants on cellular cisplatin phenotypes, the TPMT*3B and TPMT*3C haplotypes were individually created by site-directed mutagenesis and then combined to form the TPMT*3A haplotype[14]. Previous studies have reported that the TPMT* 3B, *3C and *3A variants have differential stability in cell culture and this phenotype was examined with the constructs generated in this study[21–23]. Immunodetection of TPMT variants in cell culture lysate, via an epitope tag revealed that TPMT*3C abundance was similar to wild type TPMT (*1), whereas TPMT*3B and TPMT*3A levels were reduced to 39% and 4% of wild type levels, respectively (Fig 1). The observed instability of TPMT*3B and TPMT*3A is in line with prior reports where TPMT*3A levels were virtually undetectable[21]. Similar results were observed when quantifying TPMT variant protein levels in the murine inner ear HEI-OC1 cell line (S2 Fig). These data indicate that the constructs used in this study reproduce the physiological properties associated with *TPMT* pharmacogenetic variants.

**Fig 2:**
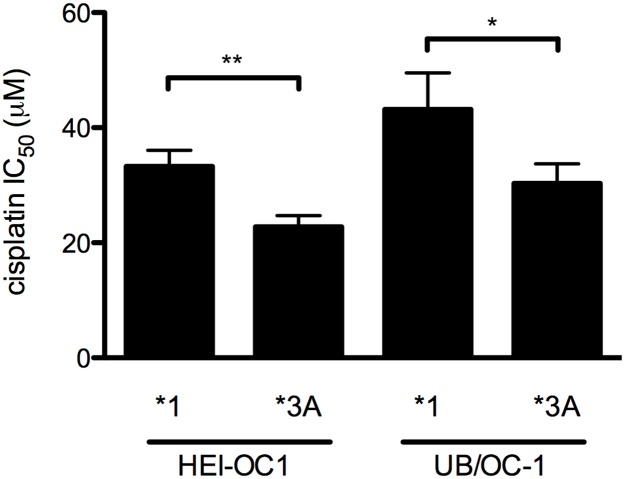
*TPMT* variation influences cisplatin cytotoxicity in murine inner ear cell lines. HEI-OC1 or UB/OC-1 cells expressing TPMT*1 or TPMT*3A (as indicated) were treated with varying concentrations of cisplatin, quantified by MTT assay and the best-fit IC_50_ values were determined using a non-linear log(inhibitor) vs. normalized response model. Dose response curves are shown in S3 Fig and curve fitting parameters are listed in S2 Table. Data are presented as the mean and standard error of the mean. * and ** denote *P* < 0.05 and *P* < 0.01, respectively using Extra sum-of-squares F test.

To examine the affect of TPMT on cellular responses to cisplatin, wild type and variant *TPMT* were transfected into two murine inner ear cells lines and a dose-response of cisplatin concentration and cell viability was established. Relative to TPMT*3A-expressing HEI-OC1 cells, viability was increased in TPMT*1-expressing cells at all cisplatin concentrations tested and this was reflected in a significantly higher cisplatin IC_50_ value in these cells as determined by MTT assay (Fig 2 and S2 Table). A moderately enhanced cell viability effect was observed when endogenous *Tpmt* was reduced by knockdown in UB/OC-1 cells that have a 3-fold lower basal expression of *Tpmt* as quantified by qPCR (Fig 2 and S2 Table). TPMT*1 particularly influenced cell viability at sub-IC_50_ cisplatin concentration where statistically significant differences were noted at 10 μM cisplatin in HEI-OC1 cells and 3.16 and 10 μM cisplatin in UB/OC-1 cells (S3 Fig). Similar observations were made in a colony survival assay performed with HEI-OC1 cells, where expression of TPMT*1 led to increased colony numbers and improved cellular morphology in this assay compared to expression of TPMT*3A (S3 Fig). These data suggest that overexpression of the functional *TPMT* allele (TPMT*1) reduces the susceptibility of murine inner ear cells to cisplatin-mediated cell death.

**Fig 3:**
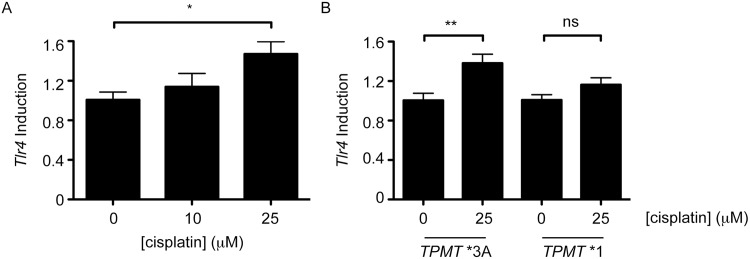
*TPMT* influences response of a cisplatin biosensor in a murine inner ear cell line. A, *Tlr4* expression was quantified in HEI-OC1 cells treated with the indicated concentrations of cisplatin. *Tlr4* expression increased with increasing cisplatin and served as a cisplatin biosensor. B, HEI-OC1 cells expressing TPMT*1 or TPMT*3A were treated with 25 μM cisplatin or left untreated and relative expression of the cisplatin biosensor was determined. Data are presented as the mean and standard error of the mean for nine replicates for untreated cells (3 independent experiments) and 6 replicates for cisplatin treated cells (2 independent experiments). *, ** and ns denote *P* < .05, *P* < .01 and not significant, respectively using one-way ANOVA with Tukey post-test analyses.

It has been previously reported that cisplatin treatment of HEI-OC1 cells leads to increased expression of the *Tlr4* gene in a dose and time-dependent manner[24]. We assessed the suitability of utilizing *Tlr4* expression as a sensitive biosensor to cisplatin using real-time quantitative PCR. As shown in Fig 3A, higher *Tlr4* expression was observed with increasing cisplatin concentrations. This *Tlr4* response to cisplatin was used to assay cells expressing wild type (TPMT*1) and inactive TPMT*3A as a sensitive measure of cisplatin exposure. TPMT*3A-expressing cells showed a significant induction in *Tlr4* expression when treated with cisplatin, whereas this was not observed in TPMT*1-expressing cells (Fig 3B). The decreased response to cisplatin in cells expressing TPMT*1 implies that these cells are exposed to a lower effective concentration of cisplatin. This finding was consistent with the mitigated cisplatin-mediated cytotoxicity observed for TPMT*1-expressing cells, and demonstrates a critical role of TPMT in cisplatin metabolism/detoxification.

## Discussion

In this work we have demonstrated that the cellular responses to cisplatin in murine inner ear cell lines are altered by pharmacogenetic variation in *TPMT*. Both cisplatin cytotoxicity and biosensor responses were increased in TPMT*3A variant-expressing cells. These altered responses suggest that TPMT plays a critical role in cisplatin metabolism and/or detoxification.

In contrast to a previous report that analyzed the correlation between *TPMT* genotype and pre-determined IC_50_ values generated in human B lymphoblastoid cells[16], the current study showed a significant impact of *TPMT* haplotype on cisplatin IC_50_ values, which were decreased by approximately one-third in the presence of TPMT*3A. This could be attributable to experimental differences since the current study employed two independent murine inner ear cell lines to generate robust cisplatin cytotoxicity data (R^2^ > 0.8, multiple independent experiments), whereas the previous study used pre-determined IC_50_ values generated in human B lymphoblastoid cells. As a point of comparison, the IC_50_ for thioguanine, a direct substrate of TPMT, was decreased by only 20% in *Tpmt* null primary astrocytes compared to wild type primary astrocytes[25], suggesting that the 30% shift in cisplatin IC_50_ values as a function of *TPMT* haplotype observed in this study is in line with other TPMT substrates.

Furthermore, indirect evidence supporting a cisplatin-*TPMT* drug-gene interaction was recently reported where the transcriptomic and metabolomic responses of cisplatin-treated ES cells were described demonstrating that cisplatin-treatment increased *TPMT* expression[26]. We similarily observed a significant upregulation of *TPMT* expression in response to 10 μM cisplatin. von Stechow and colleagues also reported that cisplatin treatment increased the concentration of the TPMT substrate, *S*-adenosylmethionine[26]. Combined with a report that SAM stabilizes TPMT[27], these observations are consistent with a putative role for TPMT in the detoxification of cisplatin. Given that increased SAM levels have been shown to exacerbate cisplatin adverse drug reactions[28] in mice, it is possible that *TPMT* variants, transcriptionally upregulated by cisplatin and destabilized by the *3A mutations, are unable to utilize the SAM co-substrate potentially leading to a two-fold mechanism of enhanced cisplatin cytotoxicity–SAM metabolite accumulation and diminished cisplatin detoxification.

It is noteworthy that mechanistic studies of cisplatin nephrotoxicity indicate that cisplatin undergoes conjugation to glutathione through the sulfhydryl moiety of the latter. *In vivo* and *in vitro* data suggest that the cisplatin-glutathione conjugate undergoes further enzymatic processing to ultimately generate a cisplatin-thiol entity that is highly reactive and nephrotoxic[29, 30]. Such a reactive thiol could act as a nucleophilic substrate bound by methyltransferases, such as TPMT, to catalyze methyl transfer from SAM and potentially detoxify the cisplatin-thiol molecule[31]. While speculative, this model would be consistent with the data observed in the current study that to our knowledge provides the first direct evidence for a cisplatin-*TPMT* interaction *in vitro*.

## Supporting information

S1 Data Source data for figures and supplementary figures. (XLSX)

S1 Fig Endogenous *Tpmt* silencing efficiency in the UB/OC-1 murine inner ear cell line. Endogenous *Tpmt* was silenced using siRNA in UB/OC-1 cell lines and relative *Tpmt* expression was determined using *Hprt1* as a housekeeping gene. A silencing efficiency of 55% was observed. Data are presented as the mean and standard error of the mean for 5 replicates in a single experiment. p-values were calculated using student T test.
(TIF)

S2 Fig Pharmacogenetic variants in TPMT show differential stability in murine inner ear cells. Representative western blot of HEI-OC1 cells expressing indicated *TPMT* variants. TPMT was specifically detected using an HA-epitope tag. The relative stability of the TPMT variants in murine inner ear cells is in good agreement with HEK293T cells (see Fig 1).
(TIF)

S3 Fig Cisplatin cytotoxicity response is influenced by *TPMT* haplotype in murine inner ear cell lines. *A*, HEI-OC1 cells expressing TPMT*1 or TPMT*3A were treated with varying concentrations of cisplatin. *B*, UB/OC-1 cells silenced for endogenous *Tpmt* and expressing TPMT*1 or TPMT*3A were treated with varying concentrations of cisplatin. Cell viability was normalized to untreated cells and quantified by MTT assay in both experiments. *C*, Colony survival assay for HEI-OC1 cells expressing TPMT*1 or TPMT*3A and grown at the indicated concentration of cisplatin. Shown are colony counts normalized to the lowest cisplatin concentration (left panel) and actual counts (right panel). The area under the curve was 200 for TPMT*1-expressing cells compared to 190 for TPMT*3A-expressing cells. *D*, Representative images of cells from colony survival assay. Data are presented as the mean and standard error of the mean for twenty replicates (4 independent experiments, panel *A*), 12 replicates (2 independent experiments, panel *B*) and 8 replicates (2 independent experiments, panel *C*). * denotes *P* < .05 using student’s T test. See S2 Table for IC_50_ and R^2^ values.
(TIF)

S4 Fig Representative full western blot images of TMPT variant expression *in vitro*. (TIF)

S1 Table Primer sequences used in this study. (PDF)

S2 Table Curve fitting parameters for cisplatin cytotoxicity studies in murine inner ear cell lines. (PDF)
