# Supplementary figures and images for: Pharmacogenetic variants in TPMT alter cellular responses to cisplatin in inner ear cell lines
Source: PLoS One. 2017 Apr 13;12(4):e0175711. doi: 10.1371/journal.pone.0175711 (PMC5391095; doi:10.1371/journal.pone.0175711)

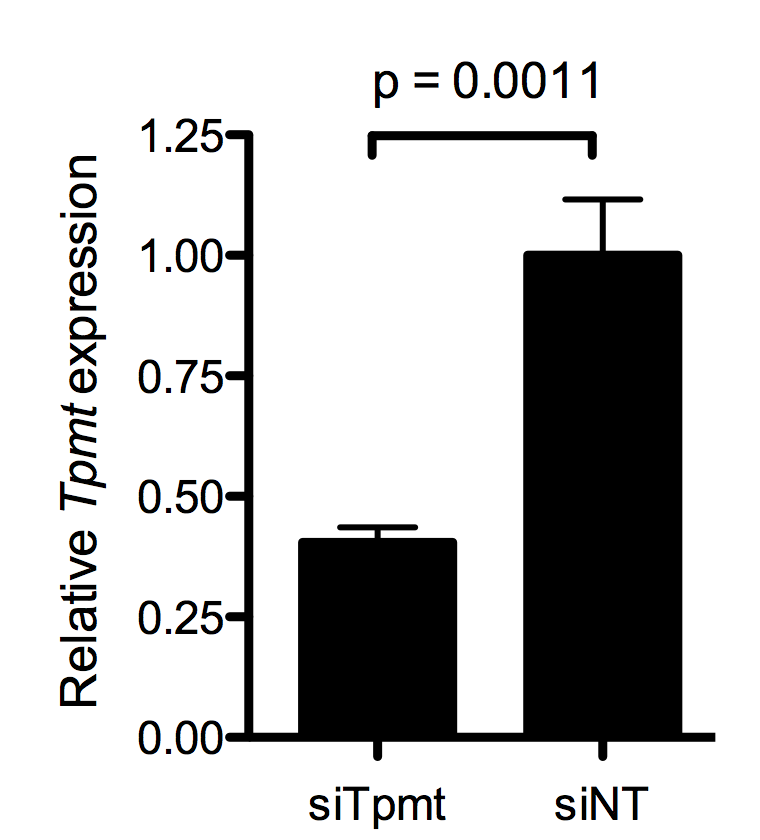

Supplement: S1 Fig — Endogenous Tpmt was silenced using siRNA in UB/OC-1 cell lines and relative Tpmt expression was determined using Hprt1 as a housekeeping gene. A silencing efficiency of 55% was observed. Data are presented as the mean and standard error of the mean for 5 replicates in a single experiment. p-values were calculated using student T test. (TIF) [file pone.0175711.s002.tif]

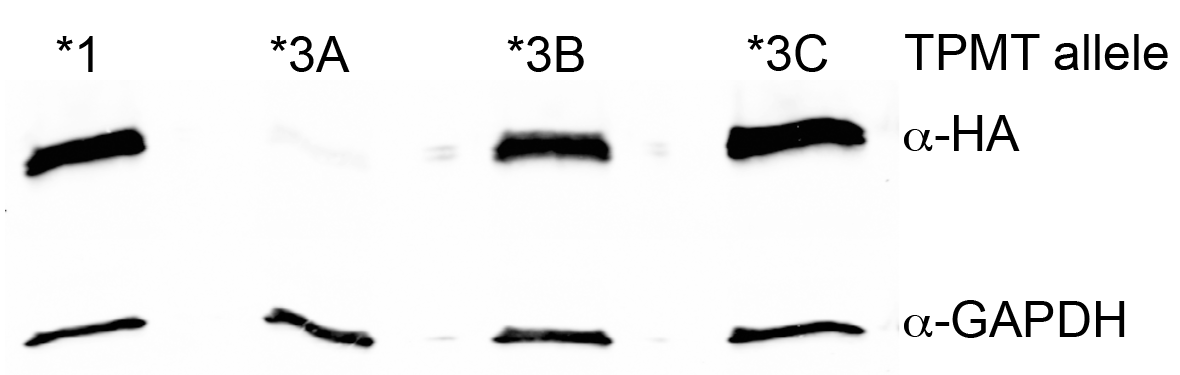

Supplement: S2 Fig — Representative western blot of HEI-OC1 cells expressing indicated TPMT variants. TPMT was specifically detected using an HA-epitope tag. The relative stability of the TPMT variants in murine inner ear cells is in good agreement with HEK293T cells (see Fig 1). (TIF) [file pone.0175711.s003.tif]

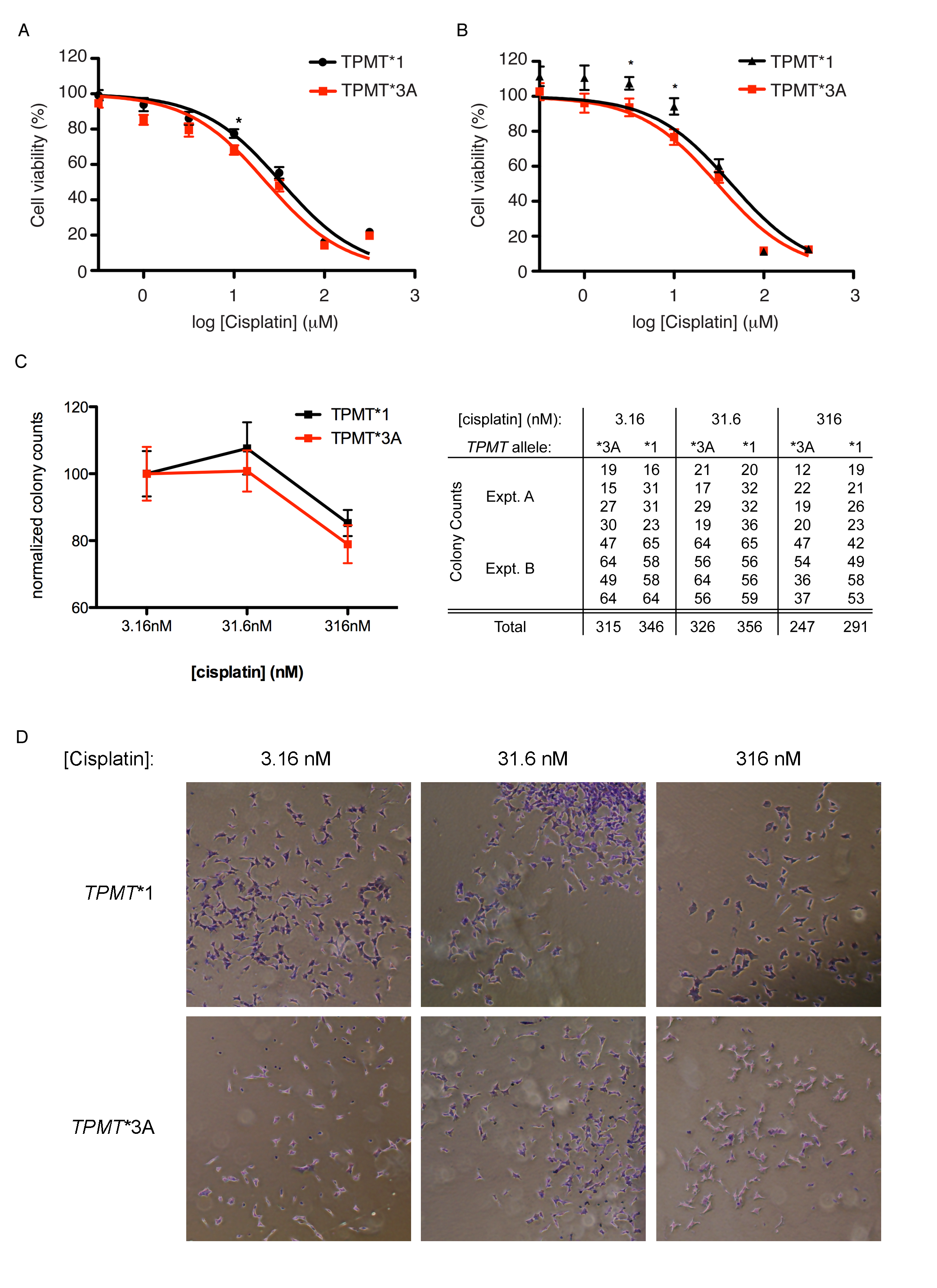

Supplement: S3 Fig — A, HEI-OC1 cells expressing TPMT*1 or TPMT*3A were treated with varying concentrations of cisplatin. B, UB/OC-1 cells silenced for endogenous Tpmt and expressing TPMT*1 or TPMT*3A were treated with varying concentrations of cisplatin. Cell viability was normalized to untreated cells and quantified by MTT assay in both experiments. C, Colony survival assay for HEI-OC1 cells expressing TPMT*1 or TPMT*3A and grown at the indicated concentration of cisplatin. Shown are colony counts normalized to the lowest cisplatin concentration (left panel) and actual counts (right panel). The area under the curve was 200 for TPMT*1-expressing cells compared to 190 for TPMT*3A-expressing cells. D, Representative images of cells from colony survival assay. Data are presented as the mean and standard error of the mean for twenty replicates (4 independent experiments, panel A), 12 replicates (2 independent experiments, panel B) and 8 replicates (2 independent experiments, panel C). * denotes P < .05 using student’s T test. See S2 Table for IC50 and R2 values. (TIF) [file pone.0175711.s004.tif]

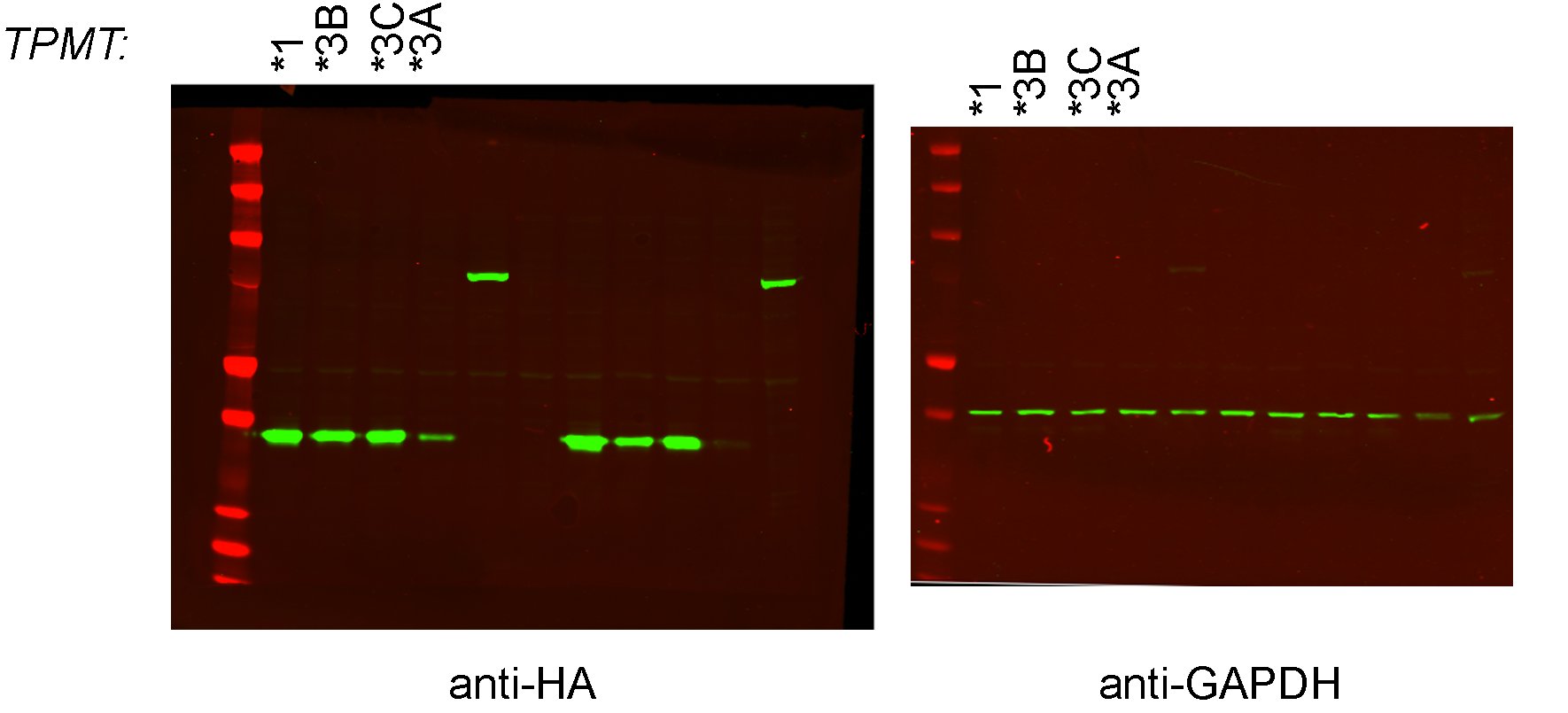

Supplement: S4 Fig — (TIF) [file pone.0175711.s005.tif]
