# Supplementary material for: Pharmacogenetic variants in TPMT alter cellular responses to cisplatin in inner ear cell lines
Source: PLoS One. 2017 Apr 13;12(4):e0175711. doi: 10.1371/journal.pone.0175711 (PMC5391095; doi:10.1371/journal.pone.0175711)
Supplement: S1 Table — (PDF) [file pone.0175711.s006.pdf]

Name	Sequence (5' to 3')
*TPMT*for1	GAGAGGATCCGATGGATGGTACAAGAACTTCAC
*TPMT*g460a_2	GGAACATTAGTTGCCATCAATCCAGGTGATCGCAA
*TPMT*g460a_as2	TTGCGATCACCTGGATTGATGGCAACTAATGTTCC
*TPMT*a719g	GGGAATTGACTGTCTTTTTGAAAAGTTATGTCTACTTACAGAAAAGTAAG
*TPMT*a719g_as	CTTACTTTTCTGTAAGTAGACATAACTTTTCAAAAAGACAGTCAATTCCC
*TPMT*t474c	GGAGCATTAGTTGCCATCAATCCAGGTGATCGCAA
*TPMT*t474c_as	TTGCGATCACCTGGATTGATGGCAACTAATGCTCC
*Tpmt*for2q	CAGGCCCACCATTTTATGTT
*Tpmt*rev2q	TCAAGACCCCAGGCTTTATG
*Hprt1*for1q	GAAGAGCTACTGTAATGATCAGTCAACG
*Hprt1*rev1q	GAGAGGTCCTTTTCACCAGCAAGC
