# Supplementary material for: Pharmacogenetic variants in TPMT alter cellular responses to cisplatin in inner ear cell lines
Source: PLoS One. 2017 Apr 13;12(4):e0175711. doi: 10.1371/journal.pone.0175711 (PMC5391095; doi:10.1371/journal.pone.0175711)
Supplement: S2 Table — (PDF) [file pone.0175711.s007.pdf]

		IC_{50} (95% CI) (μM)	*P*	n	R^2
HEI-OC1	TPMT*1	33.27 (28.25 – 39.17)	**0.0022**	160	0.83
	TPMT*3A	22.79 (19.34 – 26.84)		160	0.82
UB/OC-1[a]	TPMT*1	43.18 (32.51 –57.35)	**0.0345**	96	0.86
	TPMT*3A	30.31 (24.33 – 37.75)		96	0.81

[a] endogenous *Tpmt* expression was silenced in these cells using siRNA
